# Partition of Anammox and Nitrifiers Through Bio-Carriers for Full-Scale Sidestream Partial Nitrification–Anammox Plant

**DOI:** 10.3389/fbioe.2022.819937

**Published:** 2022-03-24

**Authors:** Jinliang Xu, Qingjie Cui, Cuina Bu, Sherif Ismail, Shou-Qing Ni

**Affiliations:** ^1^ School of Environmental Science and Engineering, Shandong University, Qingdao, China; ^2^ Suzhou Research Institute, Shandong University, Suzhou, China; ^3^ Department of Mechanical and Environmental Protection, Shandong Electric Power Engineering Consulting Institute Ltd. (SDEPCI), Jinan, China; ^4^ Environmental Engineering Department, Zagazig University, Zagazig, Egypt

**Keywords:** partial nitrification, anammox, bio-carriers, suspended sludge, biofilm

## Abstract

This study assessed the activity and community structure in different types of sludge to reveal the partition mechanism of anammox and nitrifiers in a full-scale partial nitrification–anammox plant. Batch experiments confirmed that suspended sludge had higher partial nitrification capacity, and biofilm sludge had higher anammox activity, 16.9 times higher than suspended sludge. qPCR analysis confirmed that the *amoA* gene was mainly present in suspended sludge, and the highest abundance of the *Amx* gene was observed in biofilm sludge, reaching 1.01 × 10^7^ copies/ng DNA. High-throughput results revealed that *Nitrosomonas* was the main ammonia-oxidizing bacteria with high activity in suspended sludge, and *Candidatus Brocadia* had the highest abundance of 13.4% in biofilm sludge. This is the exploration of the microbial community of three different sludge types in the full-scale sidestream PN/A system for the first time, which can guide the construction and replication of full-scale PN/A plants.

## Introduction

The development and application of the anaerobic ammonia oxidation (anammox) process has made wastewater treatment plants (WWTP) more energy-efficient in recent years. It is considered the most cost-effective biological nitrogen removal technology currently. Compared with the traditional nitrogen removal process, the process based on anammox has the advantages of less energy consumption, sludge production, and carbon sources (Van de Graaf et al., 1995; Star et al., 2007).

Anammox coupled with nitrification or denitrification has greatly promoted the engineering application of the anammox process. Partial nitrification–anammox (PN/A) is a combination of partial nitrification and anammox to achieve completely autotrophic nitrogen removal. Ammonia-oxidizing bacteria (AOB) partially oxidize the influent ammonia to nitrite aerobically. Then anammox bacteria oxidize the remaining NH_4_^+^-N to N_2_, with NO_2_^−^-N as the electron acceptor. It has been reported that the PN/A process can be successfully implemented in one-stage or two-stage reactors. The two-stage PN/A system was previously applied to real wastewater such as anaerobic digester and landfill leachate characterized by high ammonia and salinity (Ganigué et al., 2009; Lotti et al., 2019). Mosquera-Corral et al. (2005) treated the effluents from anaerobic digesters of a fish canning factory using the SHARON-Anammox system, reaching an ammonia oxidation rate of 1.0 kg N/m^3^/d. Liang and Liu (2008) investigated the effect of this coupled process on the treatment of municipal landfill leachate. Nitrification was achieved by controlling the temperature at 30°C and dissolved oxygen (DO) at 0.8–3.2 mg/L, which ultimately resulted in ammonia and total nitrogen removal rate as high as 97 and 87%, respectively. Without the pretreatment of municipal wastewater, Chen et al. (2020) achieved a two-stage PN/A through an intermittent aeration strategy in the N-SBR (sequencing batch reactor) connecting with the A-SBR. The two-stage system can be flexibly operated with high nitrogen loading and short recovery time. But there are still shortcomings in engineering application such as large area, high construction expenses, and stable issue of a standalone PN. At the same time, the effluent instability of the SHARON section impacts the nitrogen removal.

Since the affinity of nitrite-oxidizing bacteria (NOB) for DO is lower than that of AOB, low DO concentration is beneficial to the growth of anammox and also inhibits the activity of NOB. The concentration of DO below 1.5 mg/L enables the coexistence of AOB and anammox in a one-stage reactor. Many studies have confirmed that PN/A is suitable for wastewater with low C/N (Hao et al., 2002). Aquaculture and landfill leachate have been treated successfully with anammox in SBR and moving bed biofilm reactors (Molinuevo et al., 2009; Miao et al., 2014; Miao et al., 2018). Due to the long proliferation time of AOB and anammox susceptible to influent, quickly starting the PN/A system and maintaining stability have been the key issue of PN/A in engineering applications.

Recently, researchers have conducted a series of investigations into one-stage PN/A systems, including the startup time, operating modes, and parameter optimization. The performance of full-scale PN/A systems is shown in Table 1. With the in-depth study of the PN/A process, the advantages are highlighted and the scale is gradually expanded. More than 100 large-scale PN/A technologies have been applied worldwide, of which 88% of those being operated are one-stage PN/A systems (Lackner et al., 2014a).

**TABLE 1:** Performance of full-scale PN/A systems.

Reactor type	Reactor volume (m^3^)	Capacity (m^3^/d)	Type of feed water	Influent NH_4_^+^-N (mg/L)	Influent COD (mg/L)	TNE (%)	NRR (kg N/m^3^/d)	References
DEMON	2,900	1,008	Urban and industrial effluent	200	—	84	0.069	Demooij and Thomas, (2012)
CANON	600	8,400	Anaerobic digester supernatants	250	—	90	3.5	Demooij and Thomas (2012)
MBR	487	7,200–9,600	Residential and industrial wastewater	7,696,362	1,32,091	80–85	0.616	González-Martínez et al. (2021)
IFAS	15,000	—	Sludge digester filtrate	1,619	2045	86	0.17	Sy et al. (2021)
DEMON	705	—	Centrate	>500	>800	>80	0.04–0.11	Mark et al. (2014)
DEMON	2,400	—	Centrate	>1,000	1800	>90	0.54	Lackner et al. (2014b)
MBBR	832	—	Sludge liquor	1,460–1750	500–800	70	0.58	Xu et al. (2018)
CAT	384	—	Landfill leachate	634 ± 143	554 ± 97	78.9	0.5	Wang et al. (2014)
One-stage SBR	600	—	Effluent of Phospaq reactors	714	1,635	73	1.078	Abma et al. (2010)
IFAS	384	304	Landfill leachate	300–500	800–1,000	90	0.188–0.504	Huang et al. (2020)
SBR	116.6	58.3	Hydrolysis acidification effluent	136	131 ± 40	—	0.17–0.70	Fza et al. (2020)

CAT, constant aeration tank.

MBBR, moving bed biofilm reactor.

DEMON, deammonification.

IFAS, integrated fixed biofilm activated sludge.

MBR, membrane bioreactor.

CANON, completely autotrophic nitrogen removal over nitrite.

SBR, sequencing batch reactor.

TNE, total nitrogen efficiency.

NRR, nitrogen removal rate.

Although many perspectives have been explored in terms of nitrogen removal efficiency, mechanism, and population structure, few have focused on the synergistic function of microbial populations in different sludge types of the full-scale sidestream PN/A plant. In this study, bacterial activity for the suspended, biofilm, and wasted sludge (the sludge discharged from the PN/A system to maintain a proper sludge retention time) taken from a full-scale sidestream PN/A plant treating alanine production in industrial wastewater was determined in batch experiments. Combining the analysis of qPCR and high-throughput sequencing, this study investigated the microbial community variations in the suspended, biofilm, and wasted sludge and aimed to reveal the nitrogen removal mechanism in the full-scale sidestream PN/A plant, which helps understand the roles of different types of sludge and provides theoretical support for reconstruction of PN/A plants.

## Materials and Methods

### Study Site and Sample Collection

Our study site was a wastewater treatment plant, which serves the company with an annual production scale of 20,000 tons of alanine, located in Qinhuangdao HH Biological Engineering Co., Ltd., Hebei, China. The treatment capacity of the plant is 1,200 m^3^ per day. Wasted sludge was collected from the wasted sludge collection unit of the PN/A system. The microaeration caused floc sludge to be suspended in wastewater. About 100 ml of sludge–water mixture was taken from the tank, and the suspended sludge samples were obtained by sedimentation. Because of the high content of extracellular polymeric substances (EPS) secreted by anammox cells, the biofilm was attached to the bio-carriers tightly. The biofilm sludge was peeled off from the bio-carriers. Therefore, the wasted sludge, biofilm formed on the bio-carriers, and remaining suspended sludge in the PN/A tank were collected.

**FIGURE 1:**
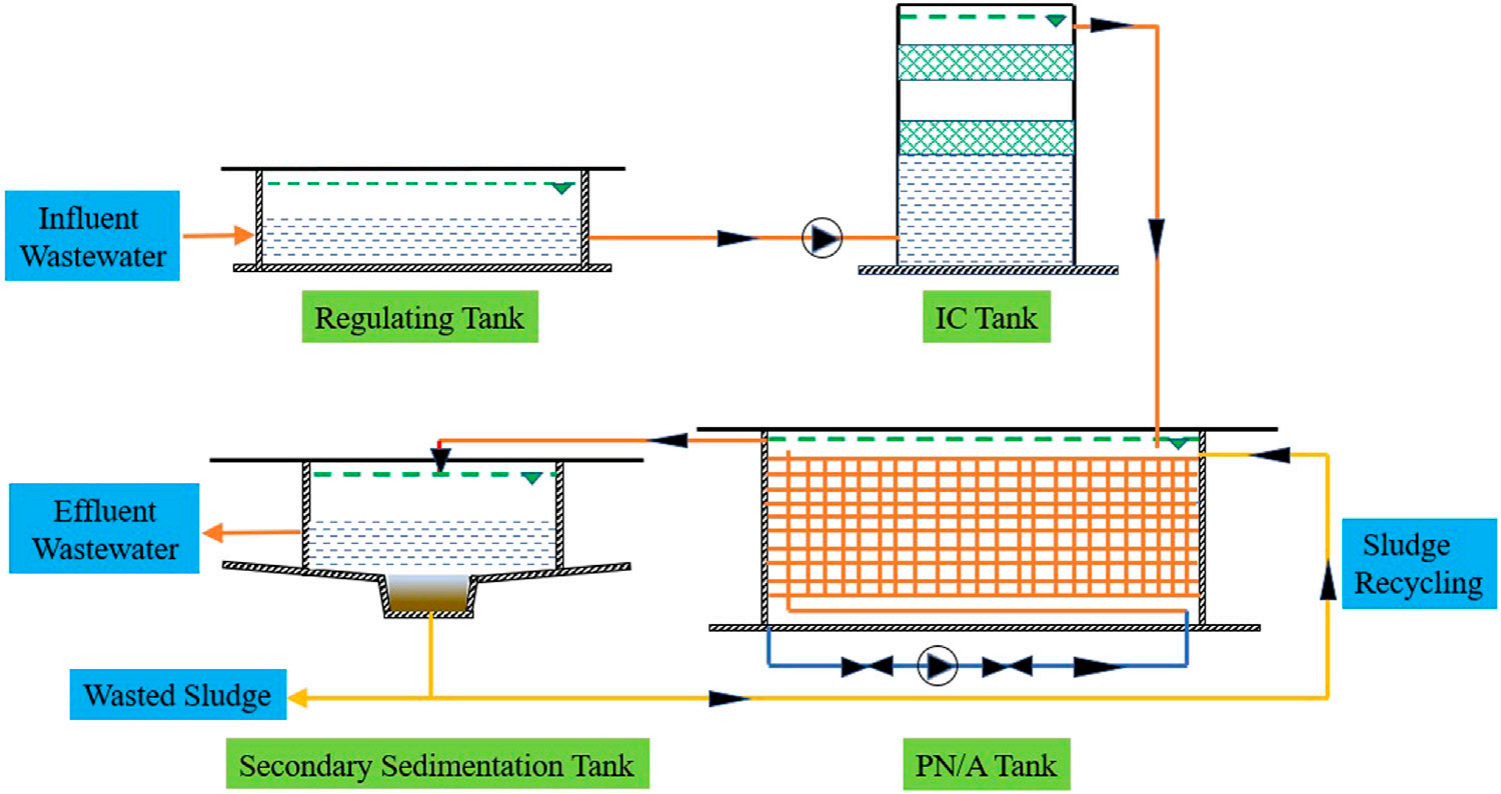
Schematic diagram of the full-scale sidestream PN/A plant. IC means internal circulation. PN/A means partial nitrification and anammox.

The wastewater was produced during alanine production with an average concentration of 8000.0 ± 14.6 mg COD/L and 700.0 ± 11.5 mg NH_4_^+^-N/L **(** Supplementary Table S1 **)**. The layout of the wastewater treatment plant is illustrated in Figure 1. Since the wastewater contained high concentration of organics, an internal circulation (IC) reactor was set up to degrade most of the COD to avoid the inhibition of anammox. The NH_4_^+^-N concentration of wastewater entering in the PN/A system was about 700 ± 11.5 mg/L, and the temperature was maintained at 25–35°C. (The temperature of the three compartments was 25 ± 2°C, and for the rest part, it was maintained at 32–35°C.) The PN/A tank was divided into six compartments, whose oxygen concentrations are shown in Supplementary Table S2. To prevent the biomass loss and improve the substrate loading, the PN/A tank was loaded with bio-carriers. The diameter of the combined bio-carriers with the plastic ring as the skeleton is about 200 mm, evenly loaded with the vinylon fiber. Finally, the average effluent COD and NH_4_^+^-N concentrations were below 350.0 ± 7.4 mg/L and 40.0 ± 3.2 mg/L, respectively (Supplementary Table S1), meeting the receiving standard of a sewage treatment plant.

### Batch Experiment for Bacterial Activity and Isotopic Tracing

In order to determine the activity of organisms in the process, the suspended sludge (S), wasted sludge (W), and biofilm sludge (B), which were taken from the PN/A tank, were tested for the activity of AOB, NOB, and anammox. The sludge samples were washed twice using 10 mM phosphate-buffered saline (PBS) to remove the substrates and then transferred to serum bottles (100 ml). In batch experiments, the AOB and NOB were fed with 500 mg/L NH_4_^+^-N and 500 mg/L NO_2_^−^-N, respectively. This experiment was conducted in a thermostatic shaker at 25°C and 180 rpm. A gas pump was used to supply air into the solution at a gas flow rate of 0.5 L/min to provide sufficient oxygen. Samples were taken within 10 h, and the concentrations of NH_4_^+^-N, NO_2_^−^-N, and NO_3_^−^-N were measured. All of the analysis was conducted in three parallel experiments.

The isotope experiment of anammox was performed in an Erlenmeyer flask (1 L) supplemented with 500 ml of medium including ^15^NH_4_^+^ as a tracer. The influent medium consisted of NH_4_^+^-N (250 mg/L) and NO_2_^−^-N (300 mg/L), KH_2_PO_4_ (40 mg/L), NaHCO_3_ (800 mg/L), CaCl_2_·2H_2_O (36 mg/L), MgCl_2_·6H_2_O (26 mg/L), and 0.5 ml trace element solution. The sludge taken from the PN/A tank was transferred to Erlenmeyer flasks after being washed three times with the PBS. The Erlenmeyer flasks were sealed with rubber plugs containing several holes to collect the gas sample. Then the headspace was flushed with 99.9% argon to remove the oxygen for 5 mins. The experiment was conducted in a thermostatic shaker at 35°C and 180 rpm. Gas samples were collected once every 2 hours by using the injection syringes from the vacuum bottles sealed with butyl rubber stoppers. The nitrogen stable isotopes were measured by Thermo Fisher Scientific, Inc., United States. Nitrogen was enriched and purified by PreCon, and the mass spectrometer was utilized to measure the ^15^N and ^14^N ratios of N_2_. The δ^15^N value was calculated by following the international standard correction.

### DNA Extraction and Quantitative Real-Time PCR

Sludge samples were collected from the PN/A tank for microbial analysis. The DNA of samples was extracted by using the PowderSoil^TM^ DNA Isolation Kit (MO BIO Laboratories, United States). The concentration of DNA was determined using a micro-spectrophotometer. Quantitative PCR (qPCR) experiments were performed to quantify the copy numbers of the 16S rRNA gene of anammox (*Amx*), the functional gene of AOB (*amoA*), and the functional gene of denitrifying bacteria (DNB) (*nirS*). As Supplementary Table S3 shows, the specific primers for anammox and AOB were Amx809F/Amx1066R (Hao et al., 2009) and amo598f/amo718r (Dionisi et al., 2002). The specific primers used for DNB were NirScd3aF/NirSR3cd (Throbäck et al., 2004). Each template was composed of a 20-μl reaction volume consisting of SYBR Premix Ex Taq (10 μl), 5 μmol/ml of forward primers (0.4 μl), 1 μl of extracted DNA, 5 μmol/ml of reverse primers (0.4 μl), and RNase-free water (8.2 μl). The tests were repeated three times to ensure accuracy. The DNA was amplified and quantified by using the Light Cycler® 480 II (Roche, Switzerland) following the thermodynamic parameters (Supplementary Table S4). And the analysis of qPCR data was based on Abs Quant/2nd Derivative Max (Zhang et al., 2016).

### High-Throughput and Microbial Community Analysis

Amplicons of 16S rRNA genes were conducted on the Illumina MiSeq platform. The total sequencing reads were stored at the Greengenes Sequence Read Archive, and Qiime v 1.8.0 was applied to handle the fastq files. To reduce the effects of unqualified sequences and improve the accuracy, the sequences of length less than 150 base pairs, primer mismatch greater than 1, and or homopolymer length greater than 8 bp were removed completely. Finally, after the valid sequencing quality controls, the average length of the sequence was approximately 395 bp.

In terms of microbial communities, the Chao1 (Chao, 1984) and ACE (Yang, 1993) indexes were adopted to reflect the richness of communities. The Shannon (Shannon, 1948) and Simpson (Simpson, 1997) indexes, which reflect the diversity of the community, were also calculated according to OTUs.

## Results and Discussion

### Nitrogen Removal and Isotope Tests

**FIGURE 2:**
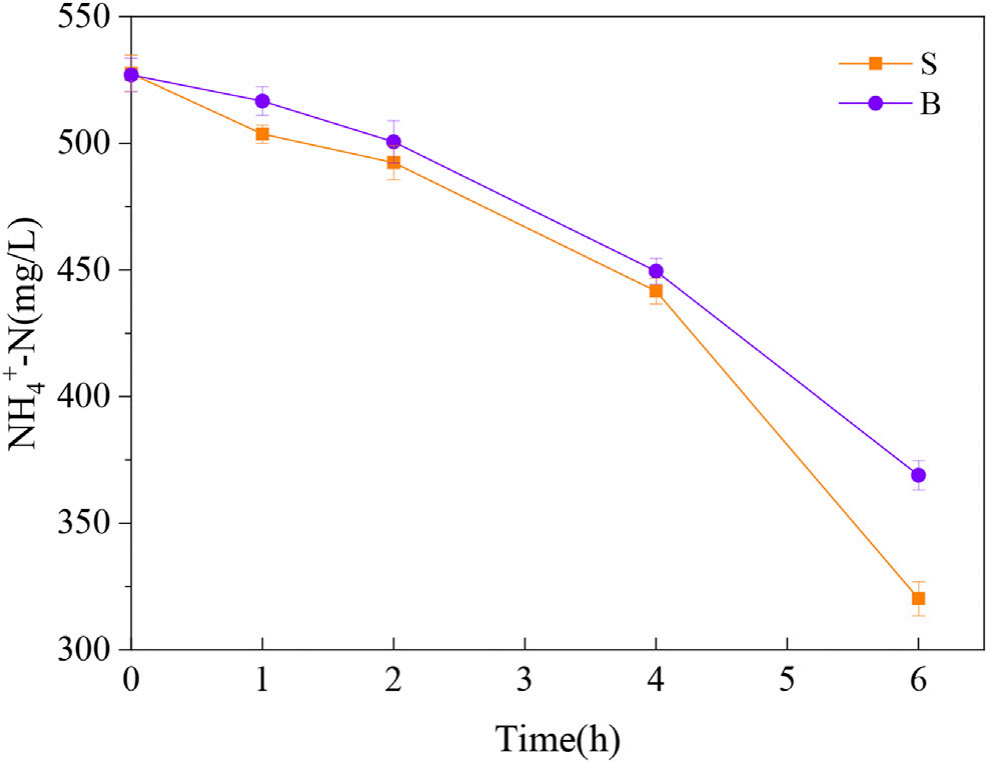
Ammonia conversion rate in S and B groups.

The substrate concentration and functional microbial activity are shown in Supplementary Figure S1, Figure 2; and Table 2. The ammonia conversion per unit time was the potential rate of ammonia oxidation by AOB. The rate of ammonia conversion was 34.55 mg/L/h in the S group and 26.34 mg/L/h in the B group, which reveals that the suspended sludge has a greater capacity for partial nitrification. Significant differences in anammox rates were observed, 8.49 μmol/L/h for biofilm sludge and only 0.50 μmol/L/h for suspended sludge. Anammox activity in the B group was 16.9 times higher than that in the S group, indicating that anammox reaction occurred mainly in biofilm sludge. Also, the nitrite concentration showed no significant variation within 10 h (Supplementary Figure S1). The rate of nitrite oxidation in the S group was slightly higher than that of the B group. But it was not enough to affect the coupling process of AOB with anammox. Therefore, partial nitrification and anammox reaction are the main pathways of nitrogen removal in this PN/A system.

**TABLE 2:** Potential activity of AOB, NOB, and anammox in S and B groups.

Group	AOB (mg/L/h)	NOB (mg/L/h)	Anammox (μmol/L/h)
S	34.55	3.80	0.5
B	26.34	1.81	8.49

### Sequencing and Alpha Diversity

**FIGURE 3:**
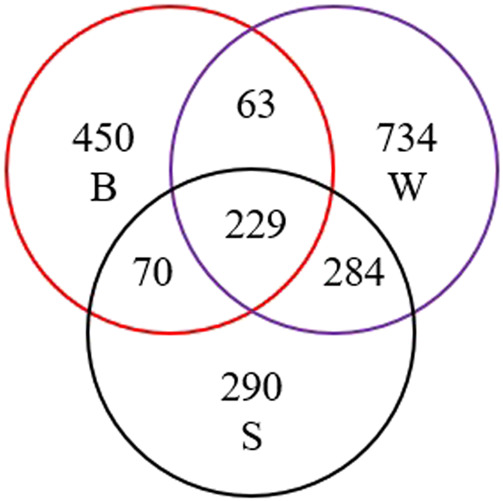
Venn diagram of OTUs in B, S, and W samples.

The V4-V5 regions of the 16S rRNA gene were amplified in the DNA extracted from different sludge samples. The PCR products were sequenced by using the pair-ended method on the Illumina Miseq. A total of 1,14,132 valid sequence reads were obtained after quality control (Table 3). Then the sequences were classified into different OTUs on the basis of their 97% level. Among the sequence reads, a total of 2,120 OTUs were obtained, of which 2,989 and 1,283 were classified into the phylum and genus levels, respectively. Among the 2,120 OTUs, 812 were from the B group, 873 from the S group, 1,310 from the W group, and 229 from all (Figure 3).

**TABLE 3:** Effective reads and alpha diversity index of three samples.

Sample	High-quality reads	Chao1	ACE	Simpson	Shannon
B	33,736	812.00	812.32	0.97	6.70
S	37,709	873.00	873.00	0.93	6.36
W	42,687	1310.00	1311.22	0.98	7.76

As shown in Table 3, the W group has the highest number of OTUs, and the B group has the lowest number of OTUs. The diversity and abundance of microorganisms were evaluated by using the alpha diversity index, including the Chao1 and ACE richness estimation indexes, and Shannon and Simpson diversity indexes. The Chao1 and ACE estimators are the highest in the W group and the lowest in the B group, which suggests that the W group has the highest abundance, and the B group has the lowest. The Shannon diversity and Simpson indexes in group W were 7.76 and 0.98, respectively, which were higher than the other two groups. It was shown that the W group had the highest community diversity and the S group had the lowest. According to the rank abundance curve, the line representing the W group is the longest and gentlest, indicating that the highest community richness and uniformity occurred in the W group.

### Microbial Community Composition

**FIGURE 4:**
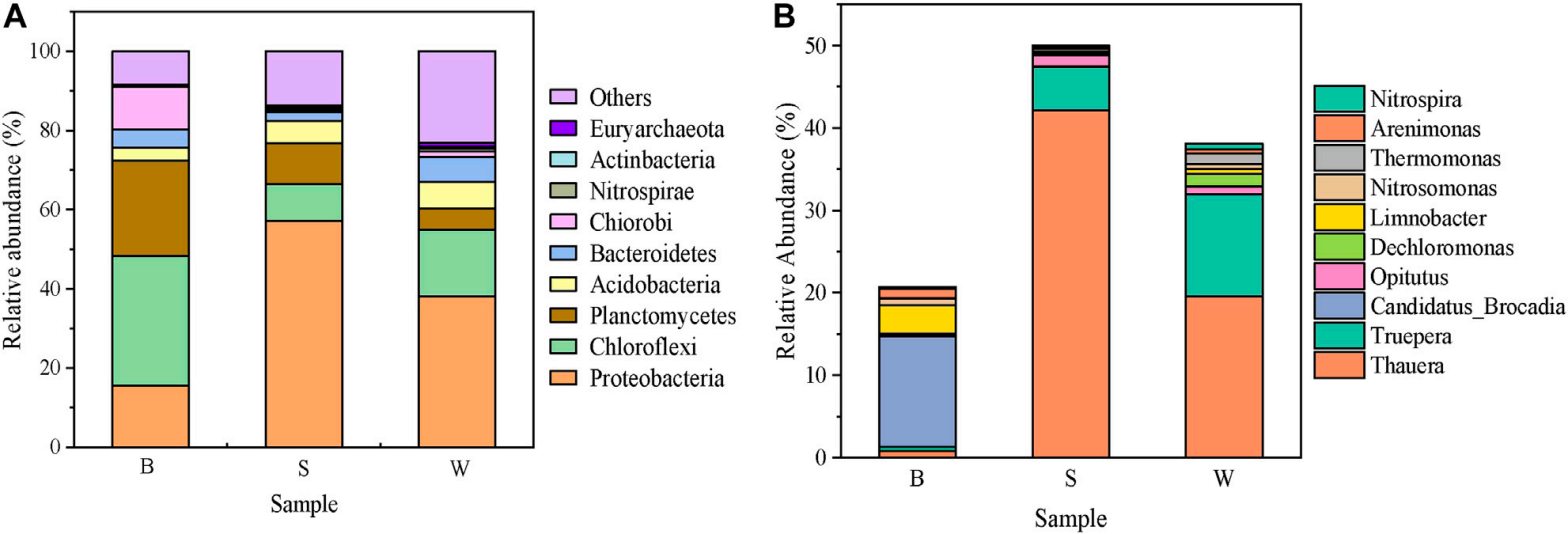
Distribution of microbes at the phylum **(A)** and genus **(B)** levels.

The community structure of three samples at the phylum and genus levels is shown in Figure 4. At the phylum level, the Proteobacteria (15.6–38.1%), Chloroflexi (9.2–32.7%), Plantomycetes (5.5–24.1%), Acidobacteria (3.2–6.6%), Bacteroidetes (2.3–6.4%), and Chlorobi (0.4–10.8%) are the predominant bacteria in all samples. Obviously, the Proteobacteria is the most abundant phylum of all the phyla identified. A small proportion of *Nitrospira* (0.2–0.7%), *Actinomycota* (0.3–0.6%), and Euryarchaeota (0.1–0.9%) are also present in three samples. At the same time, it is not difficult to find that the relative abundance of Proteobacteria in the S group is much higher than that of the other two groups. And the abundance of *planctomycetes* in the B group is more than twice that of others, accounting for 24.1%.

At the genus level, the relative abundances of anammox and DNB in three samples are quite different. *Thaurea*, belonging to the DNB, is the dominant genus in the S and W groups, accounting for 42.1 and 19.6%, respectively. But its abundance in the B group is only 0.8%. At the same time, there were lower abundances of *Nitrosomonas* and *Nitrospira* in the S and W groups. On the contrary, anammox bacteria *Candidatus Brocadia*, absent in the S and W groups, had a high abundance of 13.4% in the B group. In the adverse environment, anammox tends to grow on biofilm, rather than in suspended sludge. It has been reported that anammox bacteria tend to grow in aggregation, and biofilm is exactly one of the aggregation growth modes (Wang and Liang, 2021). Biofilm can provide anammox bacteria with a larger surface area to adhere and reduce the risk of shear forces (Cheng et al., 2022). Additionally, the developed biofilm can function as a protective barrier and create an anaerobic environment for anammox to flourish, reducing the risks caused by external changes (Liu et al., 2017). The sludge suspended in the wastewater is more easily accessible to oxygen and enriched with AOB, which is not favorable for the growth of anammox bacteria. The relative abundance of anammox in the wasted sludge is low due to insufficient uptake of ammonia and nitrite. More importantly, anammox prefers to grow tightly attached to the bio-carriers due to the high volume of EPS secreted by anammox cells. Therefore, *Nitrosomonas* and *Candidatus Brocadia* were the key functional bacteria responsible for the PN/A system, while *Nitrospira* was detected as the dominant NOB.

### qPCR Results

**FIGURE 5:**
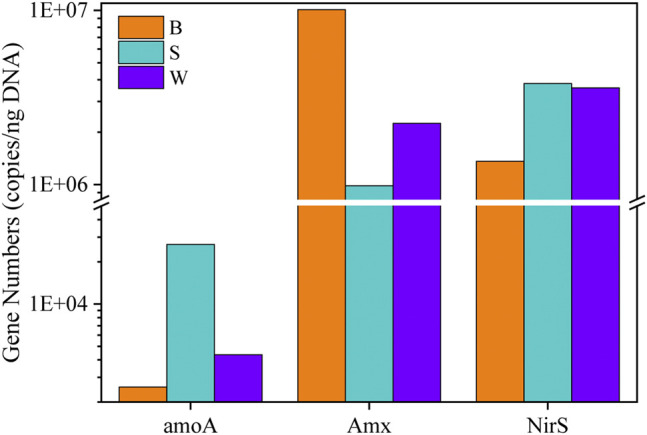
Comparison of AOB, anammox, and DNB abundance by qPCR. *amoA*, ammonia monooxygenase gene; *Amx*, Anammox; *nirS*, nitrite reductase gene.

The abundance of AOB, anammox, and DNB was detected via qPCR to evaluate the roles of various bacteria. As is shown in Figure 5, the relative abundance of functional genes of the three samples is quite different. The functional gene *amoA* has the highest relative abundance in the S group, reaching 2.66 × 10^4^ copies/ng DNA, which may be due to the stronger affinity of suspended sludge with oxygen. The copy number of *Amx* in the biofilm sludge is as high as 2.23 ± 0.139 × 10^9^ copies/ng DNA, while the copy numbers in the S and W groups are 2.71 × 10^8^ copies/ng DNA and 4.82 × 10^8^ copies/ng DNA, respectively, indicating that anammox bacteria dominated in the biofilm sludge. Actually, it can be inferred that the formation of biofilm was more conducive to the growth of anammox and the retention of biomass. Judging from the external characteristics of three samples, the biofilm sludge appeared blood red, which coincided with the aforementioned qPCR results. However, anammox bacteria also have a higher abundance in the suspended and the wasted sludge (S and W groups). It has been shown that anammox can tolerate low concentration of DO. Meanwhile, the functional gene *NirS* has a higher copy number in the S and W groups, reaching 1.04 × 10^9^ copies/ng DNA and 7.68 × 10^8^ copies/ng DNA, respectively. Correspondingly, the abundance of *nirS* in the B group was 3.80 × 10^6^ copies/ng DNA, which further confirmed that DNB existed in the PN/A tank. The low COD contained in the feed water of the PN/A system can guarantee DNB breeding, though COD was mostly removed by the IC reactor. Anammox produced a small amount of nitrate, resulting in a maximum theoretical value of total nitrogen removal of only 88%. The DNB consumed the COD and nitrate to improve the removal rate of total nitrogen (TN). However, competition between anammox and DNB makes the nitrite conversion from ammonia not fully utilized by anammox bacteria. Therefore, keeping AOB in the reactor, anammox as the dominant species, and DNB coexisting can maximize the nitrogen removal efficiency.

### Nitrogen Removal Mechanism of the PN/A System

Batch experiments confirmed the higher partial nitrification capacity in the S group. Anammox activity was much higher in the B group, and a low denitrification rate was detected. In the case of *Nitrosomonas* that performs partial nitrification (Rotthauwe et al., 1997; Gao et al., 2014), it does not occupy a high position in the PN/A tank, although the abundance in the S group is relatively high (0.8%) compared to the other two groups. High-throughput data demonstrated that anammox bacteria like *Candidatus Brocadia* were relatively more abundant in the biofilm sludge, and AOB *Nitrosomonas* was only present in the suspended sludge. It has been well established in previous studies that carriers could contribute to the enrichment of anammox bacteria and prevent biomass from loss effectively (Trigo et al., 2006; Xiaojing et al., 2015; Huang et al., 2016). The DNB *Thaurea* was mainly distributed in suspended and wasted sludge.

**FIGURE 6:**
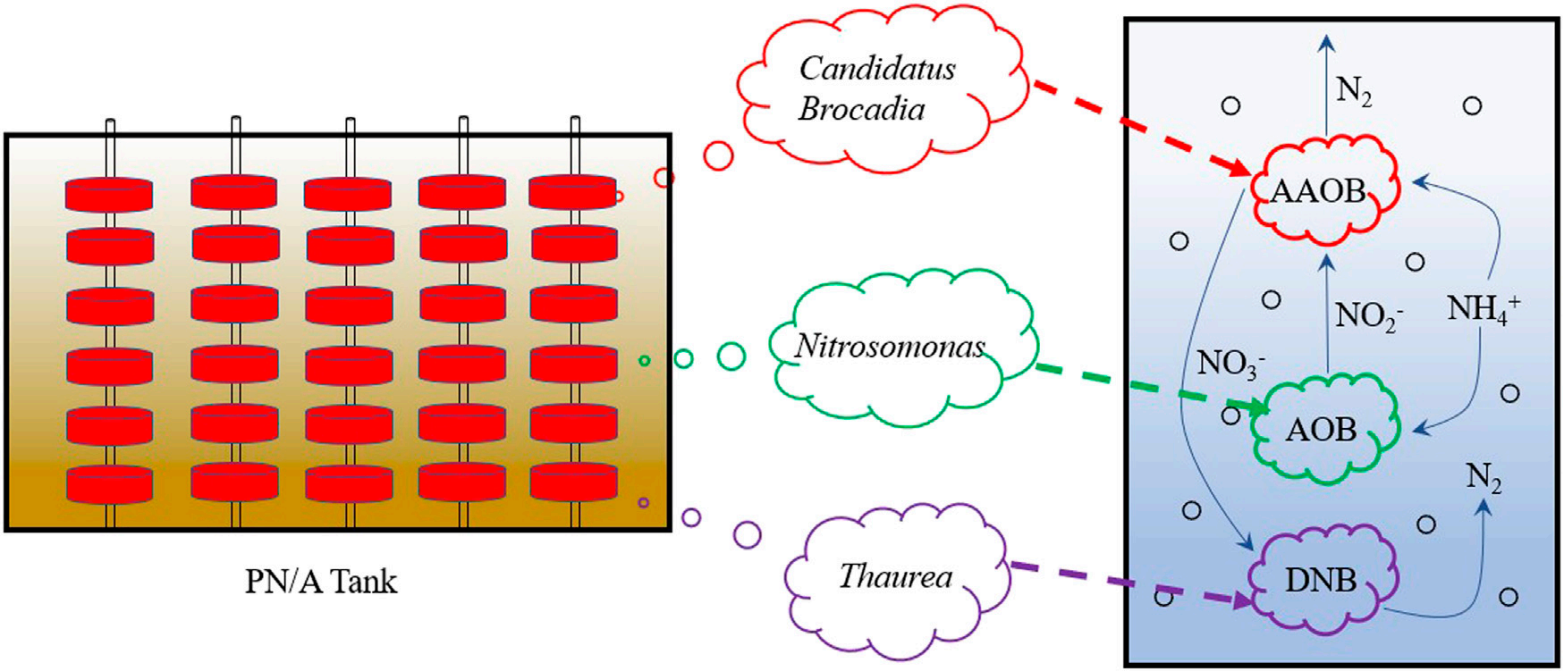
Synergistic microbial nitrogen removal process in PN/A system. The red line represents biofilm sludge, green line represents suspended sludge, and purple line represents wasted sludge. PN/A means partial nitrification and anammox.

Ammonia-oxidizing bacteria (AOB) are essential for the stable operation of the PN/A system. The first step of the process is the partial conversion of ammonia nitrogen to nitrite. In the system, despite the low abundance of AOB, their high activity can continuously provide substrate for anammox bacteria. The higher abundance of DNB, which had less effect on anammox, would promote the removal of TN and COD. Therefore, the removal of ammonia in this full-scale PN/A system primarily depends on the synergistic action of AOB and anammox bacteria (Figure 6).

### Importance of Bio-Carriers in PN/A Plants and the Significance of This Study

Ammonia-oxidizing bacteria convert ammonia into nitrite aerobically, and the suspended sludge with a larger specific surface area facilitates the affinity of AOB for DO. However, anammox bacteria tend to grow attached to the bio-carriers and form a biofilm. Biofilm plays a role in biomass retention and resistance to nitrogen loading shock. The engineered anammox process currently in operation is commonly operated in a biofilm-based bioreactor. Laureni et al. (2020) found that anammox was mainly enriched in biofilm relying on MBBR and separated from the dominant species of AOB and NOB, which confirms the important role of bio-carriers in the PN/A system. It has been reported that in unfavorable environments, anammox bacteria prefer to grow on biofilm sludge, rather than suspended sludge, which is consistent with a large number of mainstream municipal wastewater treatment systems (Hoekstra et al., 2019). The biofilm provides carriers for anammox growth and effectively prevents cells from adverse factors. Anammox bacteria attached to the bio-carriers grow to form biofilm, reducing the loss of biomass. Thus, the addition of bio-carriers causes the functional bacteria to be distributed in different sludges and achieves sludge partitioning, which is the main reason for the formation of the partial nitrification–anammox process. The project research has taken sludge morphology and functional microorganisms as the entry point, analyzed the distribution of microorganisms in different forms of sludge, and explained the importance of bio-carriers for one-stage PN/A systems more deeply. For full-scale applications in the future, the selection of bio-carriers, including the specific surface area, hydrophobicity, and cost-effective performance of the bio-carriers, may be something to consider.

## Conclusion

The PN/A system is a process that includes multiple sludge types and microbial communities. Higher potential activity of AOB was observed in suspended sludge. The anammox *Candidatus Brocadia* was mainly attached to biofilm sludge, and its activity was 16.9 times higher than that of suspended sludge. *Thaurea* had a higher abundance in suspended and wasted sludge and played a key role in nitrate removal. Synergistic nitrogen removal by multiple bacterial communities enhanced the effluent quality. Since anammox and nitrifiers prefer to attach biofilm and suspended sludge for growth, respectively, it is feasible to use bio-carriers to build a full-scale PN/A system.

## Abbreviation

AOB, ammonia-oxidizing bacteria; A-SBR, anammox sequencing batch reactor; COD, chemical oxygen demand; DO, dissolved oxygen; DNB, denitrifying bacteria; EPS, extracellular polymeric substances; PN/A, partial nitrification–anammox; N-SBR, partial nitrification sequencing batch reactor; NOB, nitrite-oxidizing bacteria; PBS, phosphate-buffered saline; TN, total nitrogen; WWTP, wastewater treatment plants

## Data Availability

The original contributions presented in the study are included in the article/Supplementary Material, further inquiries can be directed to the corresponding author.

## Abbreviation

AOB, ammonia-oxidizing bacteria; A-SBR, anammox sequencing batch reactor; COD, chemical oxygen demand; DO, dissolved oxygen; DNB, denitrifying bacteria; EPS, extracellular polymeric substances; PN/A, partial nitrification–anammox; N-SBR, partial nitrification sequencing batch reactor; NOB, nitrite-oxidizing bacteria; PBS, phosphate-buffered saline; TN, total nitrogen; WWTP, wastewater treatment plants.
