# Supplementary material for: Partition of Anammox and Nitrifiers Through Bio-Carriers for Full-Scale Sidestream Partial Nitrification–Anammox Plant
Source: Front Bioeng Biotechnol. 2022 Mar 24;10:819937. doi: 10.3389/fbioe.2022.819937 (PMC8987576; doi:10.3389/fbioe.2022.819937)
Supplement: Supplementary file 1 [file DataSheet1.docx]

**
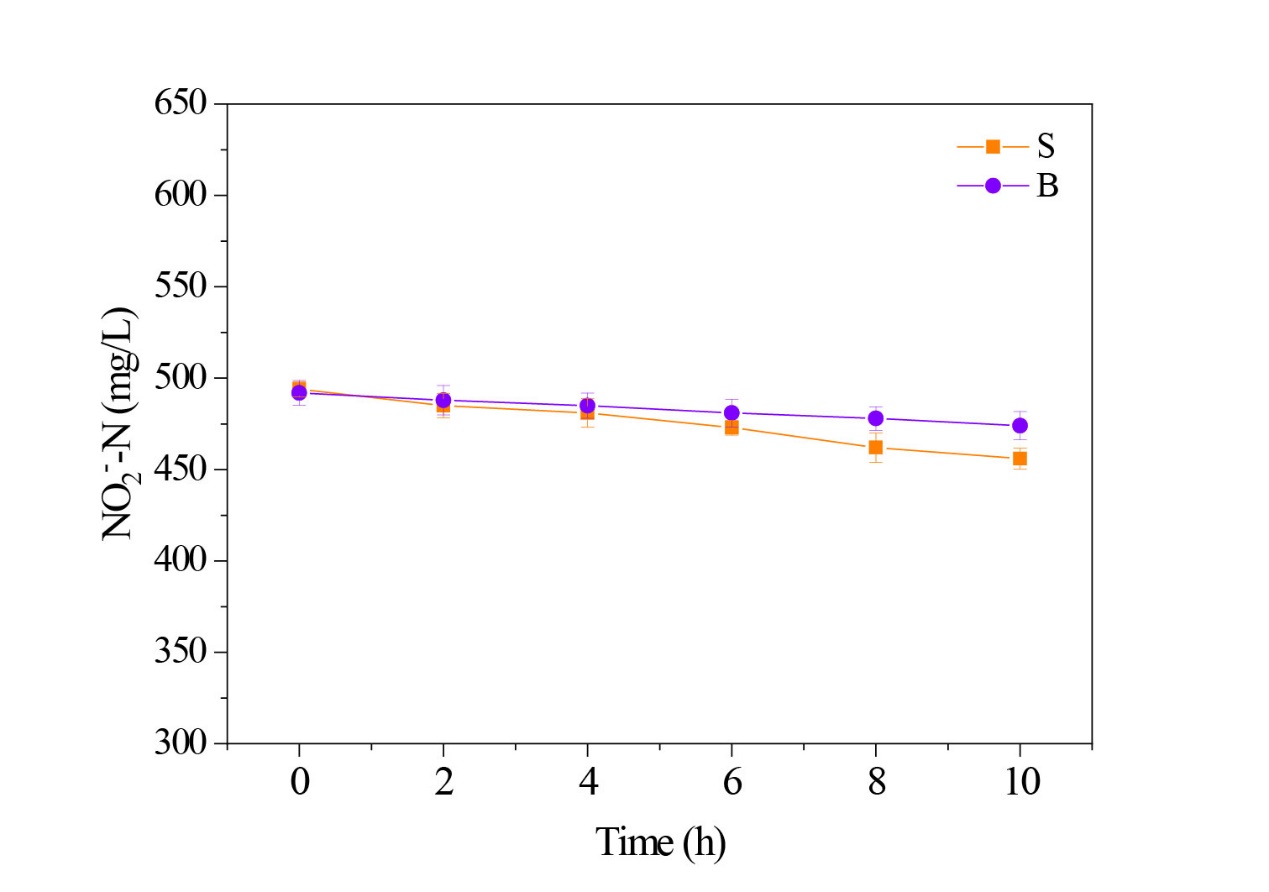
**

**Fig. S1** Nitrite conversion rate in S and B group.

**Table. S1** Characteristics of influent and effluent in the WWTP

| Index | Q | COD | NH_4_^+^-N | pH | SS | Total phosphorus |
| --- | --- | --- | --- | --- | --- | --- |
|  | (m^3^/d) | (mg/L) | (mg/L) |  | (mg/L) | (mg/L) |
| Influent | 1200.0±24.6 | 8000.0±14.6 | 700.0±11.5 | 6-9 | 600.0±13.3 | 20.0±1.2 |
| Effluent | 1200.0±19.4 | 350.0±7.4 | 40.0±3.2 | 6-9 | 220.0±9.0 | 5.0±0.8 |

**Table. S2** DO values of each compartment in PN/A tank

| DO | DO_1_ | DO_2_ | DO_3_ | DO_4_ | DO_5_ | DO_6_ |
| --- | --- | --- | --- | --- | --- | --- |
| Concentration (mg/L) | ≤0.5 | 0.5-0.8 | ≤0.5 | 0.5-0.9 | 0.9-1.5 | 1.5-2.0 |

**Table. S3 Primers used in qPCR.**

| Target gene | Specific primers | Sequence (5′-3′) | | Reference |
| --- | --- | --- | --- | --- |
| amoA | amo598f/amo718r | | GAATATGTTCGCCTGATTG  CAAAGTACCACCATACGCAG | (Dionisi et al., 2002) |
| Anammox 16S rRNA (Amx) | Amx809F/Amx1066R | | GCCCTAAACGATGGGCACT  AACGTCTCACGACACGAGCTG | (HAO et al., 2009) |
| NirS | NirScd3aF/NirSR3cd | | GTSAACGTSAAGGARACSGG  GASTTCGGRTGSGTCTTGA | (Throbäck et al., 2004) |

**Table. S4 PCR programs of target genes in qPCR.**

| Target genes | PCR procedure |
| --- | --- |
| *Amx* | Denaturation at 95 °C (30 s), 40 cycles of amplification at 95 °C (15 s), one cycle of annealing at 54 °C (30 s), and elongation at72 °C (30 s). |
| *amoA* | Denaturation at 95 °C (30 s), 58 cycles of amplification at 95 °C (50 s), one cycle of annealing at 56 °C (45 s), and elongation at 72 °C (50 s). |
| *nirS* | Denaturation at 95 °C (30 s), 40 cycles of amplification at 95 °C (15 s), one cycle of annealing at 57 °C (30 s), and elongation at 72 °C (30 s). |
